# Prion-like Protein TDP-43: Mechanisms, Diagnosis, and Therapeutic Prospects

**DOI:** 10.3390/pathogens15090890

**Published:** 2026-08-25

**Authors:** Mika Inada Shimamura, Katsuya Satoh

**Affiliations:** 1 Department of Health Sciences, Nagasaki University Graduate School of Biomedical Sciences, 1-7-1 Sakamoto, Nagasaki 852-8501, Japan; shima-m@nagasaki-u.ac.jp; 2 Multidisciplinary Pandemic Research Center, Nagasaki University, 1-12-4 Sakamoto, Nagasaki 852-8588, Japan

**Keywords:** TDP-43, prion-like propagation, amyotrophic lateral sclerosis (ALS), frontotemporal lobar degeneration (FTLD), cryo-electron microscopy (Cryo-EM), seed amplification assay (SAA), precision medicine

## Abstract

TDP-43 proteinopathies, encompassing amyotrophic lateral sclerosis (ALS), frontotemporal lobar degeneration (FTLD), and limbic-predominant age-related TDP-43 encephalopathy (LATE), represent a heterogeneous spectrum of devastating neurodegenerative disorders. For decades, the diverse clinical presentations of these diseases have complicated antemortem diagnosis and hindered the development of disease-modifying therapies. However, recent breakthroughs in basic science are beginning to address these clinical barriers, although substantial hurdles to practical clinical application remain. Structural elucidation via cryo-electron microscopy (Cryo-EM) has shattered the single-protein amyloid dogma by revealing that TDP-43 can form hetero-amyloid filaments with ANXA11, thereby providing a molecular basis for pathological strain diversity. Concurrently, the pathogenic focus has shifted toward nuclear loss of function, which triggers a systemic “RNA crisis” characterized by aberrant alternative polyadenylation (APA) and cryptic exon inclusion (e.g., *STMN2*, *UNC13A*). Crucially, this metabolic collapse is profoundly exacerbated by patient-specific genetic risk factors, acting synergistically in a “two-hit” model of neurodegeneration. To translate these findings to the clinic, next-generation diagnostic tools are emerging. Integrating neuron-derived extracellular vesicle (EV) isolation with Seed Amplification Assays (SAAs) holds promise to help overcome the structural camouflage that limits current PET imaging, potentially offering ultra-sensitive, functional strain identification in biofluids. While these structural and diagnostic milestones provide a strong foundation for precision medicine, major challenges in assay standardization and clinical validation must be addressed. Advanced therapeutic strategies—namely, splice-switching antisense oligonucleotides (ASOs) that directly restore RNA metabolism, combined with the targeted suppression of neuronal hyperexcitability—are now entering clinical trials. This review synthesizes how decoding the structural and RNA-metabolic complexities of TDP-43 is paving a promising pathway from bench to bedside, while critically discussing current translational limitations.

## 1. Introduction

The transactive response DNA-binding protein of 43 kDa (TDP-43) is not merely a bystander in neurodegeneration; it is a central executioner across a devastating spectrum of brain disorders. Under physiological conditions, TDP-43 is a tightly autoregulated nuclear protein that plays essential roles in multiple aspects of RNA metabolism [1,2].

However, in neuropathological states, it undergoes a catastrophic mislocalization, exiting the nucleus to accumulate as ubiquitinated, hyperphosphorylated inclusions in the cytoplasm of neurons and glial cells. Since its landmark discovery in 2006 as the major disease protein in amyotrophic lateral sclerosis (ALS) and frontotemporal lobar degeneration (FTLD-TDP) [3,4], cytoplasmic TDP-43 aggregates have also been identified in Alzheimer’s disease (AD), Parkinson’s disease (PD), and limbic-predominant age-related TDP-43 encephalopathy (LATE) [5,6].

The true destructive power of TDP-43 lies in its intrinsically disordered C-terminal domain (CTD), which harbors a prion-like domain (PLD) [7,8]. This specific domain facilitates nucleation-dependent polymerization, enabling misfolded TDP-43 to act as a self-templating seed that recruits and converts normally soluble molecules into toxic aggregates [9,10].

This prion-like cell-to-cell transmission is now recognized as the fundamental engine driving the spatiotemporal spread of the disease phenotype [11,12].

Despite these profound mechanistic discoveries, current clinical practice remains severely bottlenecked. Traditional diagnostic frameworks, reliant on surface-level clinical manifestations, are virtually blind to the insidious spread of TDP-43 co-pathologies, especially when masked by the classical primary features of other neurodegenerative diseases. Consequently, there is an urgent and critical demand to move beyond symptomatic assessments and detect the pathology at its molecular roots.

We are now witnessing a historical inflection point at which the meticulous elucidation of basic TDP-43 biology is directly unlocking next-generation clinical tools. The molecular decoding of how TDP-43 aggregates—specifically its unique seeding capacity, structural polymorphism, and the cascading RNA crisis caused by its nuclear depletion—provides a crucial biological framework for future clinical research, though translating these findings into robust clinical tools remains an ongoing challenge.

By understanding the physical architecture and functional properties of these propagating “strains,” we can design highly specific assays that exploit their self-replicating nature and engineer precision therapies that directly intercept their toxicity.

This review systematically discusses how to bridge the gap between fundamental molecular biology and the emerging possibilities of precision medicine, while critically addressing the current translational barriers. We first deconstruct the structural and biochemical basis of TDP-43 aggregation, highlighting the recent paradigm shift in heteromeric co-assembly and the resulting structural strain diversity [13,14,15,16,17]. Building upon this molecular foundation, we evaluate the systemic collapse of RNA metabolism and the mechanisms of cell-to-cell propagation. Crucially, we highlight how exploiting these unique seeding properties has catalyzed the development of ultra-sensitive, next-generation biomarker diagnostics such as Seed Amplification Assays (SAAs) [18].

Ultimately, we demonstrate how these deep mechanistic insights are paving a direct path toward rational, precision interventions—ranging from splice-switching RNA therapeutics to intracellular clearance strategies—designed to definitively halt the spatiotemporal spread of TDP-43 pathology [19,20,21].

## 2. Molecular Dynamics and Prion-like Propagation

Before detailing the mechanisms underlying TDP-43 spread, a clear distinction must be made between “prion-like propagation” and “classical prion infectivity.” Throughout this manuscript, prion-like propagation (or templated propagation) refers to the intercellular transmission and template-directed misfolding of pathogenic proteins, such as TDP-43, within a single organism. This mechanism drives the spatiotemporal progression of pathology throughout the central nervous system. In contrast, classical prion infectivity (or transmissible infectivity) denotes the capacity of an infectious agent to transmit disease between individuals under natural or specific exposure conditions. While TDP-43 exhibits robust experimental seeding activity in vitro and in animal models, there is currently no epidemiological evidence supporting true transmissible infectivity between humans. Therefore, to avoid conceptual confusion, we deliberately rely on terms such as “templated propagation” or “seeding activity” rather than “infectious seeding” to describe these molecular events, reserving the term “infectivity” strictly for classical prions.

### 2.1. Structural Vulnerability and the Triggers of Pathological Aggregation

As established by multiple structural studies, TDP-43 is a highly conserved 414-amino acid protein characterized by a modular architecture that dictates both its physiological functions and its pathological propensity [22,23,24]. The protein comprises an N-terminal domain (NTD, residues 1–76), two tandem RNA recognition motifs (RRM1 and RRM2, residues 106–259), and an intrinsically disordered C-terminal domain (CTD, residues 274–414).

The physiological stability of full-length TDP-43 is maintained through a delicate balance of interdomain interactions. The NTD mediates physiological dimerization, forming a head-to-tail configuration stabilized by charge complementation, which is essential for functional spatial organization [25,26,27]. Meanwhile, the RRMs govern nucleic acid binding, and NMR studies have demonstrated that transient, low-affinity contacts between the flexible CTD and the NTD/RRMs keep the protein in a dynamically autoinhibited state, preventing aberrant self-assembly [25,28].

The pathological cascade of TDP-43 is predominantly initiated by the breakdown of this structural autoinhibition, a process heavily dependent on its subcellular localization and nucleic acid interactions. In its physiological nuclear environment, the binding of TDP-43 to its specific RNA targets plays a paramount role in stabilizing the conformational folding of the RRM domains.

However, under neuropathological conditions, TDP-43 aberrantly mislocalizes to the cytoplasm, where it is largely deprived of its native RNA partners. This resulting “RNA-free” state induces a profound structural destabilization. Without the stabilizing presence of RNA, a cryptic amyloidogenic core normally buried within RRM2 (residues 247–257) becomes erroneously exposed, providing an accessible structural scaffold that significantly lowers the energetic barrier for spontaneous nucleation [29,30,31].

Concurrently, this aggregation cascade is powerfully accelerated by the aberrant phase behavior of the intrinsically disordered CTD. Under normal conditions, the prion-like CTD mediates reversible liquid–liquid phase separation (LLPS), enabling TDP-43 to dynamically assemble into membraneless organelles such as stress granules [32,33,34]. However, this delicate liquid-like state is highly vulnerable to genetic perturbations.

Disease-linked missense mutations in the *TARDBP* gene (e.g., A315T, M337V), which predominantly cluster within this region, fundamentally alter the biophysical properties of the CTD by enhancing its local hydrophobicity and intermolecular affinity. As highlighted in recent biophysical studies, these mutations act as a critical catalyst for an aberrant “liquid-to-solid phase transition” [35,36,37].

Consequently, the once-dynamic liquid droplets progressively rigidify into viscous hydrogels, ultimately maturing into irreversible, cross-linked amyloid-like fibrillar networks.

### 2.2. Fibril Architecture and the Principles of Prion-like Propagation

Ultimately, the breakdown of intramolecular networks and the irreversible phase transition allow the CTD to undergo catastrophic structural rearrangements. Groundbreaking advances in cryo-electron microscopy (Cryo-EM) and Microcrystal Electron Diffraction (MicroED) have recently revolutionized our understanding of these end-stage aggregates by resolving the atomic structures of TDP-43 amyloid fibrils [13,14,15,38].

These high-resolution studies have elegantly mapped the precise topography of the low-complexity domain (LCD), revealing that it can assemble into multiple polymorphic fibril conformations. Specifically, structural analyses have identified distinct steric zipper assemblies and polymorphic folds, notably the SegA (residues 311–360) and SegB (residues 286–331) conformers. The SegA region alone can adopt various architectures, including SegA-sym, SegA-asym, and SegA-slow forms, depending on the environmental conditions and the templating seeds [38,39].

Crucially, this remarkable structural polymorphism is not merely an in vitro artifact; it provides the physical foundation for the prion-like “strain” hypothesis in neurodegenerative diseases. It is precisely this capacity of the CTD to fold into and stably propagate distinct fibrillar architectures that is thought to dictate the heterogeneous clinico-pathological phenotypes observed across the TDP-43 proteinopathy spectrum, ranging from ALS to FTLD and LATE [5,13,14,15]. The longstanding dogma that neurodegenerative amyloids are strictly homomeric has been fundamentally overturned.

The exploration of TDP-43 strain diversity has recently entered the uncharted territory of co-assembly. Groundbreaking Cryo-EM analyses in 2024 revealed that FTLD-TDP Type C filaments do not consist of TDP-43 alone; rather, they form highly stable, heteromeric amyloid structures with an entirely distinct protein, annexin A11 (ANXA11). This historic discovery dictates that pathological strains are defined not only by intrinsic folding variations but also by their complex interactions with other host proteins.

This atomic-level complexity provides the physical basis for the disease’s macroscopic spread. The dissemination of neuronal degeneration in motor neurons during ALS progression bears a striking resemblance to prion diseases such as sporadic Creutzfeldt–Jakob disease and Gerstmann–Straussler–Scheinker disease [40,41].

Although protease-resistant PrPres and spongiform degeneration have not been reported in ALS or FTLD, the clinical onset and spatiotemporal spread of pathology are perfectly consistent with prion-like propagation [42]. The most widely accepted mechanistic model is the seeding/nucleation hypothesis, in which misfolded oligomeric TDP-43 serves as a template to recruit and convert normally folded monomers into aggregated polymers [43,44]. These structures subsequently fragment and release seeds into the extracellular space, driving cell-to-cell transmission and amplifying the disease phenotype [45].

At the molecular level, this seeding process is underpinned by a biphasic aggregation pathway involving initial oligomerization followed by a transition into high-molecular-weight aggregates. The amyloidogenic transition involves the oxidation of specific cysteine residues; sequential oxidation of Cys173 and Cys175 in RRM1 destabilizes hydrophobic interactions and drives insoluble dimer formation [46].

Furthermore, C-terminal fragment (CTF) studies demonstrate that residues 274–313 in the glycine-rich CTD are critical for aggregation [47], and truncated fragments like TDP-35 and TDP-25 can actively recruit full-length TDP-43 into neuronal cytoplasmic inclusions [48].

**Figure 1:**
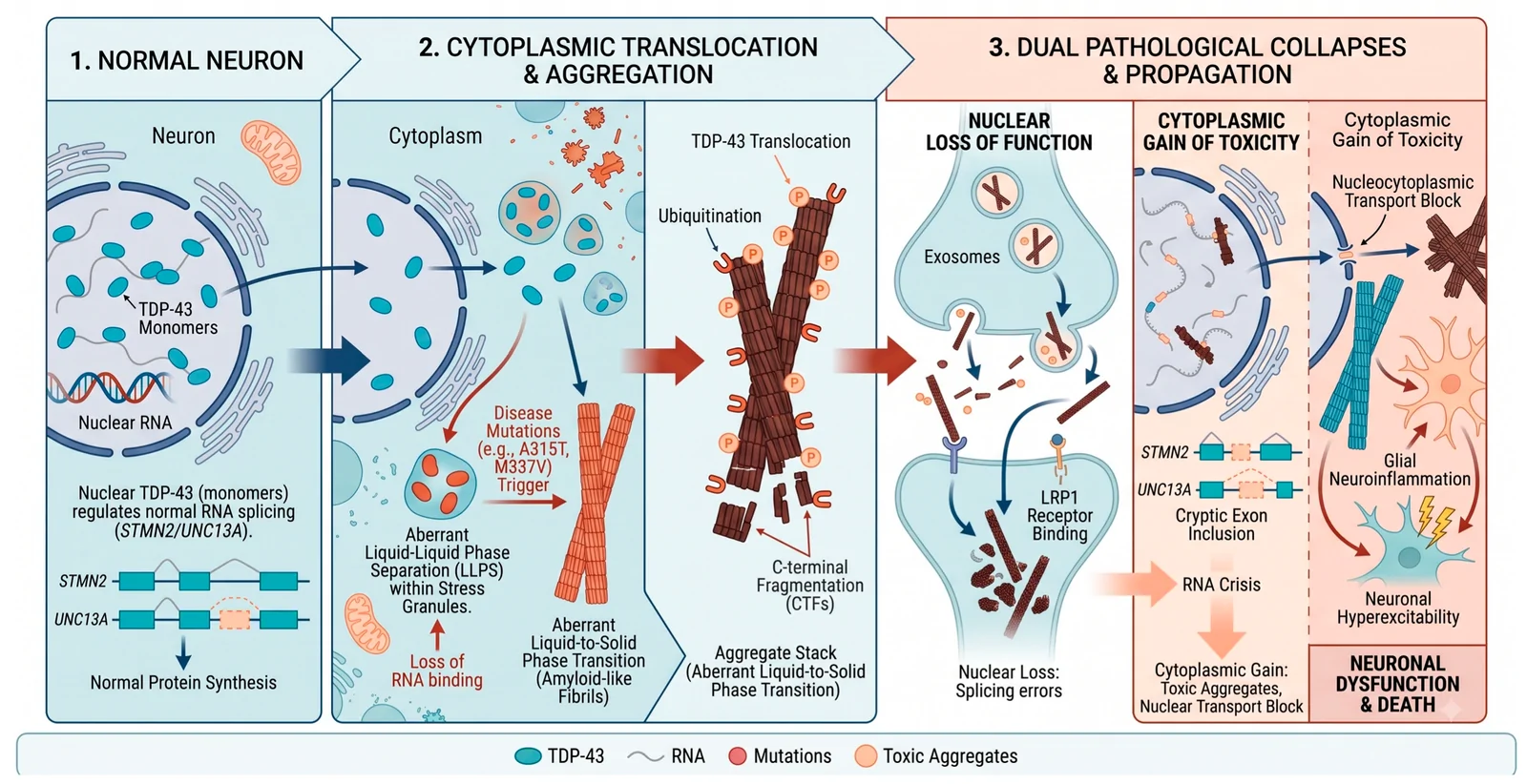
Unified Multistep Cascade of TDP-43 Pathogenesis and Prion-like Propagation. Schematic overview depicting the transition of TDP-43 from a physiological nuclear protein to pathological propagating species, leading to neurodegeneration.

Direct experimental evidence has firmly validated this seeding capacity. Fibrillar TDP-43 aggregates have demonstrated the capacity to seed the fibrillization of native TDP-43, producing intracellular aggregates with protease-resistant, Sarkosyl-insoluble cores [9]. Transfection of pathological TDP-43 extracts derived from human ALS brains into cultured cells readily induces the formation of phosphorylated, ubiquitinated cytoplasmic inclusions that proliferate between cells, establishing a serial propagation of seeds [9]. In vivo, intracerebral injections of pathogenic FTLD-TDP seeds into both transgenic and wild-type mice have successfully induced de novo TDP-43 pathology [49]. Beyond mature fibrils, oligomeric aggregates play a central role in this dissemination. Microfluidic neuronal culture studies have established the neuron-to-neuron propagation of TDP-43 oligomers via both anterograde and retrograde transsynaptic transmission [50] (Figure 1).

Critically, TDP-43 oligomers are even capable of cross-seeding Alzheimer’s amyloid-β, demonstrating an ominous interconvertibility between different amyloid species [51].

### 2.3. The “Two-Hit” Hypothesis: Genetic Risk Factors Exacerbate RNA Crisis

While nuclear TDP-43 depletion is a primary driver of RNA metabolic failure, the severity of these splicing and polyadenylation defects is heavily modulated by the patient’s genetic background, acting as a critical “second hit”. For instance, the inclusion of the cryptic exon in *UNC13A* is profoundly potentiated by specific ALS/FTD-associated risk single nucleotide polymorphisms (SNPs), such as rs12973192 and rs12608932, located within intron 20–21. These risk haplotypes act as a genetic “Achilles’ heel”; they remain silent under normal conditions but dramatically amplify cryptic exon inclusion once nuclear TDP-43 function is compromised. Similarly, widespread alternative polyadenylation (APA) changes, such as the 3′ UTR extension of *TMEM106B*, are influenced by specific genetic haplotypes, potentially involving Alu retrotransposons positioned within the risk variant. This gene–environment interaction explains the clinical heterogeneity observed in patients and underscores why individuals with specific risk alleles experience a much more aggressive disease course following TDP-43 depletion.

### 2.4. Intracellular Consequences: From Aggregation to RNA Metabolic Collapse

Once seeded, TDP-43 aggregation unfolds within a highly specific intracellular arena. Under cellular stress, the PLD of TDP-43 instigates aggregation within the dynamic milieu of stress granules [34,37]. Whether these granules act as essential precursors that generate pathogenic seeds or form independently alongside TDP-43 aggregates, their presence invariably accelerates the pathological cascade [34].

The cytoplasmic mislocalization and subsequent aggregation of TDP-43 aggressively avert its return to the nucleus, leading to a profound nuclear clearance. Pathological cytoplasmic aggregates become heavily decorated with post-translational modifications, including hyperphosphorylation, polyubiquitination, and caspase-dependent proteolytic cleavage [52,53]. Furthermore, the aberrant LLPS of TDP-43 in the cytoplasm physically inhibits nucleocytoplasmic transport, triggering the mislocalization of essential nuclear pore proteins such as Ran, RanGap1, and Nup107, ultimately culminating in neuronal toxicity and cell death [54] (Figure 1).

While the formation of cytoplasmic inclusions is a defining visual hallmark of ALS and FTLD, recent studies underscore that the true lethal blow stems from nuclear TDP-43 depletion and the ensuing collapse of RNA metabolism. The nuclear clearance of TDP-43 leads to the catastrophic mis-splicing of target mRNAs, characterized most notably by the aberrant incorporation of nonconserved cryptic exons. Crucial neuronal genes are severely compromised by this loss of function; for instance, the inclusion of cryptic exons in *STMN2* and *UNC13A* transcripts triggers premature polyadenylation, nonsense-mediated decay, and a devastating reduction in functional protein levels. Because STMN2 is vital for motor neuron repair and UNC13A is essential for synaptic vesicle fusion, their depletion directly fuels neurodegeneration.

Adding to this complex picture, recent breakthroughs have revealed a new, overarching layer of RNA metabolic failure: nuclear loss of TDP-43 triggers widespread changes in alternative polyadenylation (APA) across thousands of neuronal transcripts. When TDP-43 is depleted, cells exhibit uncontrolled usage of alternative last exons (ALEs), intronic polyadenylation (IPA), and extensive 3′ UTR extensions. A prominent victim of this disruption is *TMEM106B*, a major genetic risk factor for FTLD-TDP. TDP-43 depletion causes a significant lengthening of the *TMEM106B* 3′ UTR, which drastically reduces its translation efficiency and diminishes the levels of functional TMEM106B dimers. This newly identified APA disruption highlights a profound, systemic failure of RNA processing that exponentially exacerbates the neurodegenerative cascade. Thus, TDP-43 depletion acts as the initial domino, instigating a fatal chain reaction from splicing errors to complete translational failure.

## 3. Classification of TDP-43 Proteinopathies

The classification of TDP-43 proteinopathies has undergone a paradigm shift, evolving from simple morphological descriptions to a comprehensive, multi-layered framework. Today, the landscape of TDP-43 research is most systematically understood across four distinct dimensions: histopathological, biochemical, ultrastructural, and functional. In this review, we highlight the Seed Amplification Assay (SAA) as the crucial fourth functional tier. By elucidating how SAA-derived functional profiles correlate with the existing structural and molecular classifications, we seek to integrate these four tiers into a unified understanding of TDP-43 pathology.

### 3.1. Histopathological Classification

The histopathological classification of TDP-43 proteinopathies is based on the morphology and cortical distribution of abnormal aggregates in phosphorylated TDP-43 immunostained sections. Subtypes are determined by the relative frequency and specific cortical layer distribution of neuronal cytoplasmic inclusions (NCIs), neuronal intranuclear inclusions (NIIs), dystrophic neurites (DNs), and glial inclusions. Building upon initial criteria by Mackenzie et al. and Sampathu et al., a harmonized international consensus established Types A–D in 2011, followed by the addition of Type E in 2017 for rapidly progressive cases, formalizing the current standard classification [55,56].

**Type A:** This is characterized by numerous NCIs and short DNs concentrated in the superficial cortical layers (Layers II/III), accompanied by lentiform NIIs. It is primarily associated with behavioral variant frontotemporal dementia (bvFTD) and non-fluent variant primary progressive aphasia (nfvPPA), frequently linked to *GRN* mutations.

**Type B:** It features diffuse NCIs across all cortical layers with relatively few DNs. It is most commonly observed in ALS or FTD-MND cases and is strongly associated with *C9orf72* repeat expansions.

**Type C:** It is dominated by long, thick DNs in the upper cortical layers with relatively few NCIs, exhibiting an extremely high correlation with semantic dementia.

**Type D:** It is a rare subtype characterized primarily by numerous NIIs, exclusively associated with *VCP* mutations.

**Type E:** It is associated with older-onset, rapidly progressive FTD, featuring granulofilamentous NCIs, fine granular neuropil threads, and distinct white matter glial inclusions.

Recently, the accumulation of diffuse or granular phosphorylated TDP-43 within the neuronal cytoplasm—termed “pre-inclusions”—has been reported to precede the formation of these typical structures, potentially reflecting the earliest pathological changes [57]. However, while histopathological classification excels at mapping spatial distribution and macroscopic morphology, it is inherently limited by its inability to directly assess the underlying molecular structure or biochemical properties of the aggregates.

### 3.2. Biochemical Classification

The biochemical classification system categorizes abnormal TDP-43 extracted from the Sarkosyl-insoluble or urea-soluble fractions of patients’ brains. This is based on the Western blot (WB) band patterns of phosphorylated TDP-43, full-length TDP-43, and C-terminal fragments (CTFs). While healthy brains predominantly exhibit full-length TDP-43 at approximately 43 kDa, TDP-43 proteinopathies are characterized by the accumulation of abnormally phosphorylated TDP-43 alongside distinct CTFs at 35 kDa and 23–26 kDa.

By developing a phosphorylated TDP-43-specific antibody (pS409/410) and analyzing the insoluble fractions of FTLD-TDP and ALS brains, Hasegawa et al. revealed for the first time that the molecular weights and band patterns of these CTFs correlate closely with clinico-pathological subtypes [58]:

**Type A:** it is characterized by 23, 24, and 26 kDa CTFs, with the 23 kDa band being predominant.

**Type B:** It exhibits a distinct biochemical profile typical of ALS or FTD-MND, featuring multiple CTFs at 18, 19, 23, 24, and 26 kDa, with a dominant 24 kDa band.

**Type C:** It primarily consists of 23 and 24 kDa CTFs but conspicuously lacks the 26 kDa band, correlating with the pathological subtype of semantic dementia.

**Figure 2:**
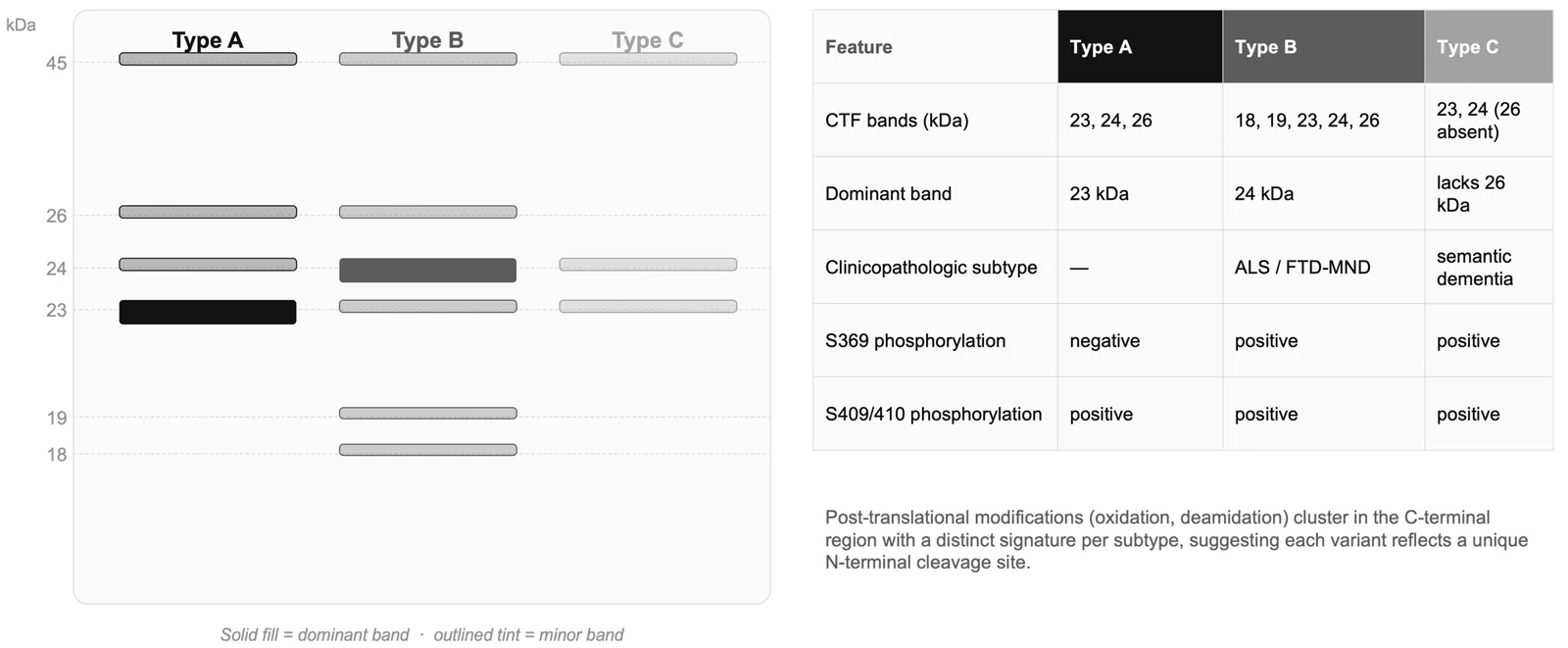
Biochemical Classification of Pathological TDP-43 Species. Schematic representation of the distinct Western blot band patterns of phosphorylated TDP-43 C-terminal fragments (CTFs) in Sarkosyl-insoluble/urea-soluble fractions extracted from patient brains (**left**). The table (**right**) summarizes the key biochemical features of neuropathological Types A, B, and C. Type A is characterized by a dominant 23 kDa band, while Type B displays a dominant 24 kDa band alongside minor 18 and 19 kDa bands. Type C is distinguished by the complete absence of the 26 kDa band. Post-translational modification profiles reveal that while S409/410 phosphorylation is universally present across all subtypes, S369 phosphorylation is positive only in Types B and C [58].

These varying band patterns are thought to reflect differences in the specific N-terminal cleavage sites of TDP-43. Furthermore, distinct disease specificities exist in post-translational modifications (PTMs). While S409/410 phosphorylation is universally observed across all subtypes, S369 phosphorylation is positive in Types B and C but largely absent in Type A. PTMs such as oxidation and deamidation are concentrated in the C-terminal region and exhibit distinct modification signatures for each subtype, strongly suggesting that each disease variant is composed of unique molecular species (Figure 2).

### 3.3. Ultrastructural Classification (Cryo-EM)

Recent advancements in cryogenic electron microscopy (Cryo-EM) have enabled the three-dimensional structural analysis of abnormal TDP-43 filaments extracted from patient brains at atomic resolution. While histopathology and biochemical profiling assess macroscopic morphology and molecular weights, Cryo-EM directly visualizes the precise physical conformation of the aggregates.

Patient-derived TDP-43 exists in the Sarkosyl-insoluble fraction as amyloid-like filaments measuring approximately 10–15 nm in diameter, presenting distinct ultrastructural morphologies dictated by the pathological subtype. Notably, Type B-derived filaments are structurally thinner than those of Types A and C. Although the filament core—primarily comprising residues G282–Q360—forms a double-spiral architecture, the specific folds and intermolecular interactions are demonstrably unique to each subtype [13,14].

These findings provide direct structural proof that the differences in CTFs and PTMs observed in the biochemical classification are physically rooted in the diverse three-dimensional conformations of pathological TDP-43. Because filaments with varying structures exhibit distinct propagation capabilities and pathogenicities, they are now understood as the physical embodiment of “TDP-43 strains”. (As detailed in the following section, this ultrastructural resolution has recently led to groundbreaking discoveries regarding heteromeric co-assembly).

As summarized in Table 1, these diverse clinico-pathological phenotypes of TDP-43 proteinopathies are intricately linked to their underlying biochemical profiles and cryo-EM ultrastructural identities.

### 3.4. Functional Classification (Seed Amplification Assay)

The Seed Amplification Assay (SAA) is a novel analytical technique that utilizes the inherent seeding activity of abnormal aggregates to induce the structural conversion of normal proteins, allowing for the ultra-sensitive detection of trace pathological TDP-43. While histopathology maps spatial distribution, biochemistry identifies molecular fragments, and Cryo-EM resolves static atomic structures, SAA fundamentally differs by evaluating the functional properties of abnormal TDP-43—specifically, its real-time seeding capacity and aggregation amplification potential.

Recent studies indicate that seeding activity, amplification rates, and the morphology of the amplified products vary significantly across different TDP-43 strains. This suggests that SAA captures dynamic differences in pathogenicity that cannot be assessed by histopathological, biochemical, or structural methods alone. Furthermore, the kinetics derived from SAA—such as reaction rate, amplification efficiency, maximum fluorescence intensity, and lag time—are highly dependent on the initial conformation and seed load of abnormal TDP-43, drawing significant attention for their robust correlation with specific disease subtypes.

In summary, the characterization of TDP-43 proteinopathies has evolved from macroscopic histopathology and biochemical profiling to atomic-level ultrastructural resolution. However, these structural and molecular assessments alone do not fully capture the dynamic, propagating pathogenicity of the aggregates. SAA brilliantly bridges this gap by evaluating the functional propagation of TDP-43 strains. Integrating this fourth functional tier with established classifications holds profound potential for elucidating disease pathogenesis, establishing definitive molecular subtypes, and accelerating the development of precision biomarkers.

## 4. The Structural Revolution: Cryo-EM Insights

The advent of cryogenic electron microscopy (Cryo-EM) has fundamentally revolutionized our understanding of TDP-43 amyloid fibrils at the atomic level. This structural resolution has provided crucial insights into how distinct pathological strains drive the diverse clinico-pathological phenotypes observed across ALS, FTLD-TDP, and LATE.

The initial breakthrough in this structural revolution occurred in 2019 when Cao et al. analyzed short fragments of the C-terminal amyloid core [38]. Their landmark study identified four distinct polymorphs—including “dagger” and “R-shaped” folds—derived from the SegA and SegB regions. By providing the first atomic evidence that TDP-43 does not assemble into a singular structure, this work definitively solidified the prion-like “strain” hypothesis for TDP-43 proteinopathies.

Building upon these early peptide-based models, the structural paradigm was significantly expanded in 2021. Li et al. successfully resolved the fibril architecture of the entire low-complexity domain (LCD, residues 267–414) at an impressive ~3 Å resolution [59]. This study revealed that the authentic amyloid core actually spans more than 100 residues, demonstrating that earlier models based on shorter fragments could not fully capture the complex topology of the aggregates. Furthermore, this comprehensive LCD model elegantly elucidated how specific ALS-linked mutations—such as A315E, Q331K, and M337V—are strategically clustered at sites critical for structural stability and intermolecular packing, offering a clear biophysical explanation for their aggressive aggregation propensity.

More recently, in 2024, Sharma et al. provided the most comprehensive structural landscape to date through their analysis of full-length TDP-43 fibrils. They identified three distinct filament architectures (Types I, II, and III) that differ significantly in their protofilament numbers and molecular assembly [60]. However, despite this extreme polymorphism, all filament types share a highly conserved structural motif termed the “amyloid key”. This pivotal finding suggests that while TDP-43 strains can adopt wildly diverse conformational folds, they maintain a universal structural anchor, effectively explaining the vast biological and clinical heterogeneity across the disease spectrum.

Despite these monumental structural advances, a critical translational challenge remains. Most current Cryo-EM structures rely on recombinant TDP-43 fibrillated in vitro. In stark contrast, authentic patient-derived aggregates are heavily decorated with complex post-translational modifications—such as phosphorylation, ubiquitination, and C-terminal truncation—and frequently engage in heteromeric co-assembly with other host proteins. Consequently, determining whether these in vitro structural models faithfully recapitulate the true in vivo pathology of the human brain is a pressing priority.

To bridge this gap, it is essential to transcend static structural snapshots. The most promising frontier lies in integrating Cryo-EM data with the dynamic, real-time seeding profiles generated by Seed Amplification Assays (SAAs). By merging atomic structural classifications with SAA-derived functional seeding kinetics, researchers can establish a comprehensive, multidimensional classification system. This synthesis of static structure and dynamic function is a vital step toward unmasking in vivo pathomechanisms, standardizing diagnostic criteria, and ultimately identifying definitive targets for precision therapeutics.

## 5. Spatiotemporal Dissemination and Translational Diagnostics

### 5.1. The Amplification Cycle: Aging, Neural Networks, and Glial Involvement

The seeded propagation of TDP-43 is not an isolated cellular event; it is a systemic cascade profoundly accelerated by biological aging. Aging actively dismantles the cellular safeguards that normally restrain protein misfolding. The age-dependent decline of the ubiquitin-proteasome system (UPS) and autophagy-lysosomal pathway (ALP) markedly impairs aggregate clearance. Simultaneously, progressive dysfunction of the nuclear pore complex (NPC) exacerbates nucleocytoplasmic transport failure, while increased mitochondrial reactive oxygen species (ROS) lower the energetic barrier for aberrant oligomerization. Together, these age-driven vulnerabilities create a highly permissive environment for TDP-43 to undergo an irreversible liquid-to-solid phase transition.

Once established, these initial pathological seeds exploit highly coordinated pathways to propagate across neighboring neurons. In vitro microfluidic models reveal that extracellular TDP-43 aggregates—secreted directly or via exosomes—are efficiently internalized by adjacent cells, largely mediated by low-density lipoprotein receptor-related protein 1 (LRP1) [9,50]. Upon cellular entry, they exert a direct “templated conversion” on endogenous TDP-43 [9,49]. This templated propagation mechanism has been rigorously validated in vivo; stereotaxic injections of human patient-derived, Sarkosyl-insoluble seeds into mouse brains induce a highly predictable, time-dependent dissemination of TDP-43 pathology [49]. Crucially, this pathology does not spread via passive diffusion but marches systematically along anatomically interconnected, functional neural pathways [61,62].

Furthermore, an emerging body of evidence dictates that this spatiotemporal spread is potently amplified by non-cell-autonomous glial mechanisms. Reactive astrocytes upregulate pro-inflammatory cytokines (e.g., IL-6, TNF-α), creating a toxic milieu that destabilizes nuclear TDP-43 in neighboring neurons [63]. Concurrently, while microglia initially attempt to clear extracellular aggregates, chronic exposure overwhelms their lysosomal capacity. This triggers an M1-like polarization where microglia inadvertently promote the dissemination of partially degraded, seed-competent TDP-43 fragments via exosomes [64,65]. Recognizing this glial-driven neuroinflammatory amplification loop is vital, as it propels the pathology beyond direct transsynaptic connections and presents a compelling rationale for immunomodulatory therapies.

### 5.2. Overcoming Structural Camouflage with Seed Amplification Assays

The realization that pathological TDP-43 propagates systemically as distinct conformational strains has catalyzed a paradigm shift in in vivo diagnostics. Historically, confirming TDP-43 proteinopathy in living patients was hindered by profound clinical overlap with Alzheimer’s disease and other tauopathies. Recently, significant translational efforts have focused on developing specific positron emission tomography (PET) tracers, such as [18F]ACI-19626, engineered to selectively bind TDP-43 inclusions [66].

While these first-generation PET tracers represent a monumental leap for in vivo imaging, they are fundamentally constrained by a profound structural limitation: they consistently fail to detect FTLD-TDP Type C pathology. The recent Cryo-EM breakthroughs provide the exact physical explanation for this diagnostic blind spot. Because Type C filaments exist not as pure TDP-43 homomers, but as complex heteromeric co-assemblies with ANXA11 [15], the specific conformational pockets required for PET tracer binding are physically concealed. This steric occlusion acts as an effective “structural camouflage”, rendering the pathological aggregates invisible to conventional ligand-binding imaging techniques.

This mechanistic reality exposes a critical vulnerability in relying solely on static structural recognition for diagnosis, underscoring the importance of complementary fluid-based, functional biomarker platforms—most notably, the Seed Amplification Assay (SAA). Unlike PET tracers that depend on accessible surface pockets, SAA fundamentally bypasses this structural camouflage by evaluating the dynamic, functional properties of the aggregates. By capturing the inherent self-templating “seeding activity” of pathological oligomers actively released into the cerebrospinal fluid (CSF) or blood, SAA utilizes these trace proteins as biological amplifiers in vitro.

We are now entering a new era of diagnostics in which antemortem monitoring is increasingly feasible. Based on preliminary data, advanced SAAs may theoretically be capable of identifying the conformational strain actively proliferating within a patient’s biofluids, which could eventually help unmask co-pathologies in living individuals; however, this strain-resolving capability has not yet been clinically validated. Concurrently, the degree of nuclear loss of function can be dynamically assessed by detecting *STMN2* and *UNC13A* cryptic RNAs and peptides isolated from blood or CSF-derived extracellular vesicles (EVs). Combining SAA-based seeding activity with these cryptic markers represents a promising strategy for overcoming the limitations of structural camouflage and may ultimately support the development of precision therapeutics, pending further clinical validation.

### 5.3. Overcoming Detection Barriers: The Role of Extracellular Vesicles (EVs)

While Seed Amplification Assays (SAAs) offer transformative potential for in vivo diagnosis, the clinical reality is that the absolute concentration of pathological TDP-43 in biofluids like cerebrospinal fluid (CSF) and plasma is exceedingly low and often masked by complex matrices. To overcome this significant technical hurdle, the frontier of biomarker development has shifted toward the isolation and enrichment of neuron-derived extracellular vesicles (EVs) from peripheral fluids. Cutting-edge consortia (such as the DZNE collaboration) are leveraging recent technological advancements to isolate these neuronal EVs [67], providing a concentrated, CNS-specific “liquid biopsy.” This EV-enrichment not only enhances the SAA-based detection of TDP-43 aggregates but also enables the ultra-sensitive quantification of downstream loss-of-function markers such as cryptic exons and peptides (e.g., HDGFL2) [68]. Integrating SAA with EV-isolation technologies represents the most viable path toward robust, non-invasive patient stratification and target engagement monitoring in clinical trials.

While SAA holds significant promise for neurodegenerative disease diagnostics, its clinical application is currently constrained by several major limitations, and establishing robust clinical evidence remains a critical challenge for the future. Current hurdles include the lack of assay standardization across different laboratories, the extremely low abundance of pathogenic seeds in accessible biological fluids, and confounding matrix effects that can alter amplification efficiency. Furthermore, there may be structural and conformational differences between experimentally amplified aggregates and the authentic aggregates present in patient brains. Until these methodological and biological limitations are resolved through large-scale, longitudinal clinical validations, the routine use of SAA for individualized strain identification and antemortem monitoring remains an experimental frontier rather than an established clinical reality.

## 6. The Precision Medicine Era: Therapeutic Strategies Targeting TDP-43

### 6.1. The Paradigm Shift: From Palliative Care to Precision Medicine

The robust identification of TDP-43 propagation and structural strain diversity provides a transformative framework for therapeutic intervention. Historically, the treatment of TDP-43-associated neurodegenerative disorders has been purely palliative. However, profound advances in elucidating the molecular pathogenesis of TDP-43—specifically its nucleocytoplasmic mislocalization, liquid-to-solid phase transitions, cascading RNA crisis, and prion-like intercellular transmission—have inspired a new era of rational drug design.

As we transition into an era where advanced fluid biomarkers, such as Seed Amplification Assays (SAAs), enable the in vivo detection and strain-subtyping of propagating seeds, the therapeutic paradigm is firmly shifting towards precision medicine. The emerging therapeutic landscape has evolved from broadly targeting cellular stress into a powerful hybrid strategy: repairing the foundational RNA metabolic defects (the offensive front) while simultaneously intercepting and degrading toxic cytoplasmic strains (the defensive front).

To provide a balanced perspective on this rapidly evolving field, the following subsections categorize current interventions strictly by their stage of development: established clinical evidence, ongoing clinical trials, and preclinical or conceptual approaches.

### 6.2. Established Clinical Evidence

Currently, there are no established disease-modifying therapies that specifically halt the prion-like propagation of TDP-43 in humans. The current standard of care for ALS and FTD remains primarily symptomatic, centered on Riluzole (an antiglutamatergic agent) and Edaravone (a free radical scavenger). While Riluzole extends survival and Edaravone slows functional decline in specific subpopulations, these standard-of-care medications offer only modest delays in progression without directly reversing the underlying proteinopathy or its prion-like propagation mechanisms [69,70]. Thus, clinically validated interventions that directly target the disease process itself remain highly limited.

### 6.3. Ongoing Clinical Trials

Recognizing the specific downstream RNA targets of TDP-43 loss of function has catalyzed the rapid development of precision gene therapies, particularly splice-switching antisense oligonucleotides (ASOs). Instead of merely managing downstream symptoms, these precision ASOs—such as QRL-201 and Trace Neuroscience’s *UNC13A*-targeting candidates—are designed to fundamentally correct splicing aberrations at the RNA level. By sterically hindering the splicing machinery from recognizing the cryptic exons in *STMN2* and *UNC13A* pre-mRNAs, these ASOs successfully redirect the process toward normal, mature transcripts, aiming to rescue synaptic and neuronal dysfunctions.

**Targeting Neuronal Hyperexcitability**

In addition to correcting RNA splicing defects and clearing protein aggregates, addressing the downstream electrophysiological consequences of TDP-43 proteinopathy is a critical therapeutic pillar. A classic and prominent pathophysiological feature of ALS is the severe hyperexcitability of motor neurons, which directly contributes to excitotoxicity and rapid neuronal death. Recent precision medicine strategies aim to directly counteract this hyperexcitability. For example, targeted therapies are being developed to correct the function of ion channels (such as the Kv7.2/7.3 potassium channel opener, QRL-101, developed by QurAlis) to dampen motor neuron hyperexcitability and prevent excitotoxic damage. By stabilizing the electrical activity of neurons, these therapies provide a highly synergistic approach to RNA-repairing ASOs, forming a comprehensive strategy that protects the motor neuron from both internal metabolic collapse and external excitotoxic stress.

**Targeting Neuroinflammation:** Complementing these approaches, inhibition of the MAPK pathway has emerged as a promising anti-inflammatory strategy: the MEK inhibitor Trametinib suppressed immune overactivation and mitigated TDP-43 toxicity in a Drosophila model, and multiomic profiling of human ALS tissue has independently highlighted the MAPK pathway as a therapeutic target [71,72].

**Combating Hyperexcitability and Metabolic Failure:** Agents such as Mexiletine (a sodium channel blocker) and Perampanel (an AMPA receptor antagonist) are being repurposed to prevent motor neuron hyperexcitability and excitotoxicity [73,74]. Meanwhile, drugs like Metformin and the combination AMX0035 (sodium phenylbutyrate and taurursodiol) were designed to address mitochondrial dysfunction and endoplasmic reticulum stress. While AMX0035 initially demonstrated a significant slowing of functional decline in the Phase 2 CENTAUR trial and received conditional approvals, it subsequently failed to show statistically significant efficacy in the larger, randomized Phase 3 PHOENIX trial, leading to its voluntary withdrawal from the global market. This major clinical setback underscores the formidable challenges of translating early-stage clinical signals into sustained therapeutic benefits for ALS patients [75].

**Drug Repurposing via iPSC Screens:** High-throughput screening using ALS-derived iPSCs has identified candidates like Ropinirole (a dopamine agonist) and Bosutinib (a Src/c-Abl inhibitor), both of which are actively advancing through clinical trials for their neuroprotective effects [76].

Beyond RNA- and inflammation-directed strategies, small molecules such as PU-AD (an epichaperome inhibitor targeting Hsp90) and planar aromatic compounds (e.g., mitoxantrone) are advancing in clinical trials, aiming to prevent the aberrant accumulation of RNA-binding proteins in stress granules [51,77].

### 6.4. Preclinical and Conceptual Approaches

In parallel, viral therapies and gene editing offer permanent corrective potential. Adeno-associated virus (AAV)-mediated delivery of CRISPR/Cas9 components or RNA interference (RNAi) constructs targeting *TARDBP* represents a promising avenue for TDP-43-linked ALS therapy [78]. Additionally, ADAR2-mediated A-to-I RNA editing—which catalyzes the mRNA deamination of adenosine into inosine—is being explored as a direct means to correct aberrant TDP-43 function and prevent the excitotoxicity driven by unedited AMPA receptors in sporadic ALS [74].

Complementing RNA repair, a formidable cellular defense strategy focuses on halting the spatiotemporal cell-to-cell spread of pathological seeds. A cutting-edge approach involves AAV-delivered intrabodies (e.g., Mabylon), which are engineered to directly intercept and degrade toxic TDP-43 strains within the cytoplasm before they can propagate.

Broadening this defensive front are agents that robustly induce autophagy. Rapamycin (Sirolimus), an mTOR inhibitor, not only promotes the autophagic clearance of TDP-43 aggregates but also activates immunosuppressive T regulatory cells (Tregs), countering neuroinflammatory amplification loops [79]. Other promising autophagy inducers currently under investigation include Tamoxifen [80], Colchicine [81], and IMS-088, a semisynthetic analog of withaferin-A that has been shown to decrease TDP-43 pathology and restore cognitive performance in transgenic mice [82].

Beyond direct RNA and aggregate targeting, an extensive pipeline of therapies aims to protect the neuronal microenvironment from TDP-43-induced toxicity.

**Modulation of Phosphorylation and DNA Repair:** Aberrant hyperphosphorylation promotes cytoplasmic mislocalization. IGS-2.7, a novel CK-1δ inhibitor, has successfully reduced TDP-43 phosphorylation and neuroinflammation in transgenic models [83]. Additionally, strategies targeting DNA double-strand break (DSB) repair mechanisms seek to restore genomic stability compromised by nuclear TDP-43 depletion [84].

### 6.5. Toward Integrative Polytherapy

Given the multifaceted nature of TDP-43 pathology—encompassing RNA splicing failure, protein aggregation, metabolic collapse, and neuroinflammation—it is increasingly clear that monotherapies may be insufficient. The future of TDP-43 therapeutics undoubtedly lies in combinatorial approaches (polytherapy). Integrative frameworks, such as the MEND protocol and multi-target herbal formulations (e.g., GRAPE formula or berberine) [85], have been primarily discussed and investigated within the Alzheimer’s disease literature. While some preliminary preclinical evidence suggests a potential relevance of compounds like berberine to TDP-43-related pathways, these integrative approaches should not be presented as an established clinical consensus for TDP-43 proteinopathies. Instead, they serve as useful conceptual paradigms, highlighting that successfully addressing the multifaceted TDP-43 spectrum will likely require multi-targeted, synergistic interventions that simultaneously repair the nucleus, clear the cytoplasm, and quiet the inflammatory glial environment.

## 7. Conclusions and Future Perspectives

Over the past two decades, TDP-43 has emerged from relative obscurity to be recognized as the central executioner across a devastating spectrum of neurodegenerative diseases. However, as outlined in this review, we are no longer merely observing its destruction; we have finally deciphered its molecular blueprint. The revelation that TDP-43 drives disease progression through prion-like, cell-to-cell propagation has fundamentally shifted our understanding of neurodegeneration from a static cellular failure to a dynamic, spreading pathology.

The recent structural revolution has provided profound mechanistic insights into this spatiotemporal spread. By resolving the atomic architecture of polymorphic fibril conformations—and uncovering the complex heteromeric co-assembly with proteins like ANXA11—we now understand the physical basis of TDP-43 strain diversity. Crucially, this atomic-level understanding has directly catalyzed the development of functional, fluid-based diagnostics. By overcoming the structural camouflage that evades conventional imaging, next-generation Seed Amplification Assays (SAAs) have equipped us with the unprecedented ability to detect, amplify, and subtype these pathological seeds in living patients.

Consequently, the therapeutic landscape is experiencing a monumental paradigm shift. While early clinical candidate developments like AMX0035 initially provided hope, its subsequent market withdrawal underscores the volatile nature of translational efforts, and rapamycin’s dual mechanism of promoting autophagic clearance while modulating neuroinflammation positions it as a robust candidate; however, this is only the beginning. We are now standing at the precipice of the precision medicine era.

To definitively conquer TDP-43 proteinopathies, future research must aggressively drive forward across four interconnected frontiers:

**Integrating Structure and Function:** We must comprehensively map the precise mechanisms of TDP-43 strain diversity, fusing static Cryo-EM structural data with dynamic SAA kinetics to decode how specific polymorphs dictate clinical heterogeneity.

**Deploying Antemortem Precision Diagnostics:** The field must accelerate the clinical implementation of ultra-sensitive fluid biomarkers—combining SAA-derived strain identification with the detection of cryptic RNAs, phosphorylated TDP-43, and neurofilament light chain (NfL) in blood and CSF—to unmask co-pathologies at their earliest stages.

**Pioneering Integrative Polytherapy:** Recognizing that monotherapies are insufficient against a cascading pathology, we must engineer combination therapeutic strategies that simultaneously address the three pillars of the disease: repairing nuclear loss of function (via splice-switching ASOs), halting aggregate propagation (via intrabodies or targeted clearance), and quieting the neuroinflammatory environment.

**Advancing Translational Disease Models:** We must continue to refine patient-derived iPSC models and transgenic systems to ensure they faithfully recapitulate the complex, human-specific nuances of TDP-43 co-assembly and systemic RNA metabolic failure.

While clinical care has historically been primarily supportive, the development of targeted, disease-modifying interventions represents a promising direction for future medicine. However, translating these profound insights into definitive therapies remains a challenging, long-term goal that requires overcoming substantial methodological and clinical hurdles.

Finally, as we look toward the future of TDP-43 precision medicine, recognizing the distinct roles of glial cells in aggregate clearance and neuroinflammation will be essential [86,87]. Elucidating how patient-derived TDP-43 strains propagate in vivo [49] and exhibit neurotoxic effects reflecting different disease progression rates [88] will further inform our understanding of spatiotemporal dissemination. Moreover, targeting exosome-mediated clearance pathways [89], intercepting cytoplasmic phase separation events [90], and correcting fundamental toxic cascades initiated by C9orf72 expansions [91,92] will provide critical therapeutic avenues. Combining these multi-targeted approaches will be paramount in definitively halting the progression of TDP-43 proteinopathies.

## Figures and Tables

**Table 1:** Structural and biochemical classification of TDP-43 strains.

Feature	Type A	Type B	Type C	Type D	PLS-TDP (Emerging)
Associated Clinical Phenotypes & Genetics	bvFTD, nfvPPA (e.g., *GRN* mutations)	ALS, FTD-MND (e.g., *C9orf72* mutations)	Semantic Dementia (SD) (Sporadic)	IBMPFD (*VCP* mutations)	Primary Lateral Sclerosis (PLS) (Sporadic)
Neuropathology (Morphology & Distribution)	Abundant crescentic/oval NCIs and short DNs (predominantly in superficial cortical layers)	Diffuse NCIs across all cortical layers and moderate DNs	Abundant long, thick DNs (predominantly in upper cortical layers) and few NCIs	Numerous lentiform neuronal intranuclear inclusions (NIIs) and short DNs	NCIs and DNs (morphologically resembling Type A pathology)
Biochemical Profile (Major CTF Band Pattern)	Predominant 23 kDa band	Predominant 24 kDa band	23 and 24 kDa bands (lacking 26 kDa band)	~23, 24, and 26 kDa bands (similarly to Type A/B)	Distinct 17 and 22 kDa bands
Cryo-EM Ultrastructure (Strain Identity)	“Chevron fold” (unique structural polymorph)	“Double-spiral fold” (unique structural polymorph)	Heteromeric filaments (TDP-43 + ANXA11 co-aggregation)	Not yet resolved	Heteromeric filaments (TDP-43 + ANXA11 co-aggregation)

Abbreviations: bvFTD, behavioral variant frontotemporal dementia; nfvPPA, non-fluent variant primary progressive aphasia; MND, motor neuron disease; IBMPFD, inclusion body myopathy with Paget disease of bone and frontotemporal dementia; NCI, neuronal cytoplasmic inclusion; DN, dystrophic neurite; NII, neuronal intranuclear inclusion; CTF, C-terminal fragment. This table summarizes the diverse clinico-pathological phenotypes and neuropathological distributions of TDP-43 proteinopathies, illustrating their precise correlations with underlying biochemical profiles and recent cryo-electron microscopy (cryo-EM) ultrastructural identities. Distinct fibril architectures—such as the Chevron fold in Type A, the double-spiral fold in ALS/Type B, and TDP-43/ANXA11 heteromeric filaments in Type C and primary lateral sclerosis (PLS)—act as molecular determinants of disease phenotypes.

## Data Availability

No new data were created or analyzed in this study. Data sharing is not applicable to this article.
